# The Inflow, Throughput and Outflow of COVID-19 Patients in Dutch Hospitals: Experiences from Experts and Middle Managers

**DOI:** 10.3390/healthcare12010018

**Published:** 2023-12-21

**Authors:** Lidy Okkerman, Dennis Moeke, Stan Janssen, Jeroen van Andel

**Affiliations:** Research Group Logistics & Alliances, HAN University of Applied Sciences, 6802 EJ Arnhem, The Netherlands; dennis.moeke@han.nl (D.M.);

**Keywords:** hospitals, COVID-19, patient flow logistics, lessons learned, best practices

## Abstract

At the beginning of 2020, the large and unforeseen inflow of COVID-19 patients had a deep impact on the healthcare operations of Dutch hospitals. From a patient flow logistics perspective, each hospital handled the situation largely in its own particular and improvised way. Nevertheless, some hospitals appeared to be more effective in their dealing with this sudden demand for extra care than others. This prompted a study into the factors which hindered and facilitated effective operations during this period. We provide an overview of actions and measures for organizing and managing the inflow, throughput and outflow of COVID-19 patients within Dutch hospitals from various types of departments in a large number of hospitals in The Netherlands, based on interviews with nine experts and twelve hospital managers. Ten actions or measures have been identified, which have been divided into the following three dimensions: Streamlining of the underlying in- and external processes, reducing unnecessary or undesirable inflow of patients and increasing or making more adequate use of the available (human) capacity. The main lessons learned are the importance of integral tuning in the care process, giving up habits and self-interest, good information provision and the middle manager as a linking pin.

## 1. Introduction

**Figure 1:**
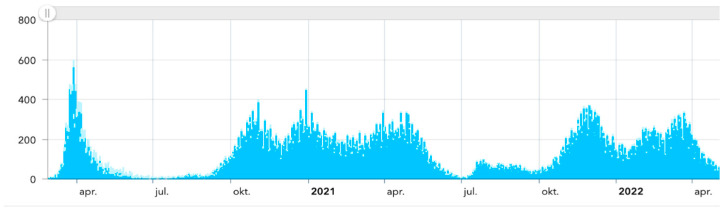
Total number of COVID-19 hospital admissions in The Netherlands [2].

At the onset of the COVID-19 pandemic in early 2020, most Dutch hospitals were deeply affected by the highly contagious coronavirus (i.e., SARS-CoV-2), which can cause lethal respiratory problems. In 2020, from 1 March to 31 December alone, about 34.000 COVID-19 patients were admitted to Dutch hospitals [1]. Figure 1 shows the total number of Dutch COVID-19 admissions during the period of March 2020–April 2022 [2]. It is clear from the figure that hospitals faced multiple “COVID-19 waves”. The unparalleled influx of COVID-19 patients created a serious challenge in terms of patient flow logistics, where patient flow logistics is defined as the following: *Ensuring the delivery of the right care, at the right time, in the right place, by the right person, where what is ‘right’ is based on a trade-off between the needs and preferences of the individual patient, the professional responsibility of the healthcare professional(s) and the efficient use of available resources* [3].

In order to make the best use of scarce available Intensive Care Units (ICUs) and to prevent the risk of infection, regular (non-acute) care was scaled down. According to Central Statistics Office Netherlands [4], in 2020, Dutch hospitals admitted nearly 230.000 fewer patients than a year earlier. This corresponds to a 12 percent decrease from the previous year. At the same time (for some of the hospital departments), the workload increased dramatically. For example, ICUs of a large number of hospitals reached their maximum occupancy, forcing patients to be spread across other hospitals in The Netherlands and, in some cases, even Germany [5].

Nearly all Dutch hospitals faced this same complex challenge, namely, optimally absorbing a large and strongly fluctuating inflow of highly contagious COVID-19 patients with a high probability of a rapidly changing health situation. From a patient flow logistics perspective, each hospital handled the situation to a large extent in its own way. This prompted a qualitative study, whose main results are presented in this paper. In it, we provide an overview of actions and measures for organizing and managing the inflow, throughput and outflow of COVID-19 patients within Dutch hospitals from various types of departments in a large number of hospitals in The Netherlands, based on interviews with nine experts and twelve middle managers (see also Table 1). This shows the recommended courses of action or steps along with user experiences.

The remainder of this paper is structured as follows: Section 2 provides an overview of related work. Next, in Section 3, we describe the methodology applied to carry out this study. The results are presented in Section 4, a discussion is described in Section 5 and the paper ends in Section 6 with an overview of the main conclusions.

## 2. The Existing Literature

Numerous studies have been published in response to the COVID-19 pandemic. However, only a limited number of these studies focus on lessons learned by hospitals from an organizational perspective. This conclusion is supported by the study of [6]. They report that, amongst 11 Belgian hospital, only a limited number of healthcare teams reflected on the lessons learned and on the maintenance of good practices that came from the COVID-19 pandemic. As a result, “*opportunities for healthcare innovation and quality improvement seemed to be missed*” [6]. In our opinion, this observation makes studies in which lessons learned are presented and discussed especially valuable. Hence, “*a crisis such as the COVID-pandemic can destabilize organizations but can also act as a catalyst for improvement and innovation*” [7].

Below, we provide an overview of some example studies in which lessons learned are shared. In the paper of [8], nine lessons learned from the COVID-19 pandemic for improving hospital care delivery are presented. Among other things, they provide an overview of elements to include in a hospital disaster plan for dealing with an increased volume of patients or workforce shortages. Ref. [9] explored the lessons learned by hospital managers in Sweden with regard to staff management during the COVID-19 pandemic. The results of their study indicate that the pandemic created four types of challenges relating to staff management: staff movement within hospitals; the addition of external staff; the addition of hours for existing staff through overtime and new shift schedules; and the avoidance of lost hours due to sickness or fatigue. Lessons learned from a healthcare facility that was set up within a short time frame to attend to the convalescent needs of a large number of COVID-19 patients in the early phase of handling the pandemic are presented by [10]. Regarding the deployment of remote monitoring systems during the COVID-19 pandemic, ref. [11] offers insights and strategies for implementing remote monitoring programs at scale and improving the sustainability of these efforts. There are also many papers which describe the experiences of healthcare workers during the COVID-19 crisis [12,13,14,15,16]. For instance, ref. [17] identified interventions to strengthen healthcare workers’ capacity to cope with acute stress caused by healthcare pressure during the first COVID-19 wave.

And although several scientific studies focused on the organizational lessons learned by hospitals during the COVID-19 pandemic, to the best of our knowledge, no scientific studies have been published with a focus on the organization and management of the inflow, throughput and outflow from a hospital-wide perspective. However, there are studies that focus on (the optimization of) specific parts of the patient flow in the context of the COVID-19 pandemic (see, e.g., [18,19,20,21]).

When it comes to improving the performance of healthcare systems, patient flow logistics plays a crucial role (see, e.g., [22,23,24,25,26]). This involves not only performance improvement in terms of efficiency, but also, for example, reducing waiting time [27] and achieving a more proportional distribution of the workload of involved healthcare professionals [28]. According to the well-known Little’s Theorem [29], from an aggregated perspective, the average departure rate of patients (i.e., the patient flow) of a steady-state system depends on the:

1. Average lead time (i.e., the average length of stay);
2. Amount of work in progress (i.e., the average number of patients in the system).

Hence, Little’s Theorem states that under general conditions, in the long run, the average departure rate of patients (µ) is equal to the average number of patients in a hospital (L) divided by the average length of stay (W). As such, the mathematical formula of the departure rate is rather straightforward: µ = L/W.

When taking this formula as a starting point, there are roughly three ways in which the inflow, throughput and outflow can be improved, namely:

1. Streamlining of the underlying in- and external processes.
2. Reducing unnecessary or undesirable inflow of patients.
3. Increasing or making more adequate use of the available (human) capacity.

The streamlining of the underlying processes can, for example, be achieved by standardizing procedures (see, e.g., [30,31]), the elimination of unnecessary and non-value adding activities (see, e.g., [32]), reducing artificial variability (see, e.g., [30,33]) and/ or improving responsiveness (see, e.g., [34,35]). This can involve processes within the hospital (internal processes) as well as beyond the walls of the hospital (external processes). Furthermore, less unnecessary or undesirable inflow of patients will reduce the workload of the system (see, e.g., [36,37]). Finally, expanding or making smarter use of the available capacity also has a direct positive impact on the workload (see, e.g., [38,39]).

## 3. Methods

This article describes a study conducted among experts in patient flow logistics and middle managers in hospitals. The experts involved had functions as an expert, consultant or manager of a support division. A support division provides support to the daily healthcare operations. The experts advice managers on how to organize. The middle managers held positions such as team leader, head of department or healthcare manager. The middle managers can assess the feasibility of provided advice by using their experience with organizational measures (see Table 1).

Most middle managers working in the primary process of Dutch hospitals frequently began their working life as a healthcare professional, and their task is to monitor and ensure the quality of daily operations within the set budget [40]. The middle manager is in the organizational position between healthcare professionals and the board of directors.

During the fall of 2020, interviews were conducted among nine experts. Each of the interviews lasted 35–40 min and were audio-recorded. Seven of these interviews were conducted face-to-face and two via an e-mail questionnaire. The were conducted according to the principles of semi-open interviews with the experts. The questions focused on the main bottlenecks that they encountered in practice and tips, actions and measures on how hospitals could improve the inflow, throughput and outflow of COVID-19 patients. Interviews were recorded and transcribed on paper. The interview data were systematically analyzed, summarized and discussed within the research team involved. The researcher annotated text excerpts from the interviews with codes. After classifying the codes, linkages between these groups were established [41]. The research team reviewed and discussed this strategy. Based on the content of the interviews, themes have been identified, along which the actions and measures have been classified.

In addition, between March 2022 and May 2023, twelve interviews were conducted with middle managers in various types of hospitals (see Table 1). The managers were interviewed face-to-face at the respective hospital location. Each of the interviews lasted 45–60 min and were audio recorded. To support the interviews, so-called vignettes (see, e.g., [42]) were used, where vignettes in this case involved examples of hypothetical situations in which choices must be made regarding patient flow logistics. The interviews were analyzed with atlas.ti version 9. The researcher coded text fragments in this case as well, structured the codes and created relationships between them, and the research team evaluated and analyzed the findings [41].

The research was carried out in accordance with Dutch legislation for healthcare institutions (WMO, WBGO, AVG). We conformed to the Sector Protocol for Research Quality Assurance 2016–2022 and the Dutch Code of Conduct for Research Integrity 2018.

## 4. Results

In this results section, we present an overview of the main actions and measures. The structure of this results section is based on the three-way division mentioned at the end of Section 1. Table 2 shows, for each of the aspects, a summary of the identified actions and measures.

### 4.1. Streamlining of the Underlying in- and External Processes

#### 4.1.1. Improving ICU Transfer and Discharge

When the condition of a COVID-19 patient worsens during their stay at a regular nursing ward, it is discussed with the patient, the family and the pulmonologist whether a transfer to the ICU is useful. Several managers indicated that possible negative consequences are discussed openly and honestly during this meeting (for example: long-term artificial respiration under very difficult circumstances, no or hardly any visitors and possible transfer to another hospital). Experience shows that, due to this open and honest discussion, some patients choose to not to be transferred to the ICU. One of the managers also came up with an example in which the cardiac monitoring (CCU) was being used as a step-down facility in order to speed up the ICU discharge of COVID-19 patients. Regarding the set-up of a CCU, the mentioned manager said the following: “If the IC starts to overflow with a capacity demand, then you start thinking together: How can we support it?”

#### 4.1.2. COVID-19 Cohort Wards

COVID-19 patients were brought together in cohort units within a department, or a separate cohort department was set up. There were no two streams mixed together with the risk of contamination. For the cohort department, there were separate measures, which were clear for the workers. Working in a department with COVID-19 patients was tough for healthcare workers. Working in protective clothing is very uncomfortable and reduces personal recognizability. The latter resulted in less good contact with colleagues and patients. The measure of some middle managers was to rotate employees in this department and non-COVID-19 departments. This did require more time for employees to change when entering and leaving and led to more use of protective equipment. A middle manager of a very large hospital had the impression that employees who did not work in a COVID-19 department had no idea of the hectic environment there. This miscommunication and subsequent learning opportunity led to the creation of an educational film about the COVID-19 department for all hospital staff members.

#### 4.1.3. COVID-19 Triage Tent with CT Scanner

One of the managers indicated that a triage tent was set up at the entrance of the hospital to perform a CT scan on patients with COVID-19 symptoms. This approach avoids disrupting the regular process at the emergency department (ED).

#### 4.1.4. Remote Healthcare and eHealth Applications

Two hospitals made use of a mobile digital application which makes it possible, under certain conditions, to discharge COVID-19 patients from the hospital earlier and to continue their recovery at home. The application makes it possible to remotely monitor the health condition of the patient. Important measurement data (body temperature, oxygen saturation and any complaints) are shared with a special home monitoring team on a daily basis. Because measurements take place regularly, any deterioration in the patient can be detected at an early stage, and acute readmissions are prevented as much as possible. Via the app, patients can also be remotely supported in reducing oxygen.

### 4.2. Reducing Unnecessary or Undesirable Inflow of Patients

#### 4.2.1. Adequate Transition Management

Before sending a COVID-19 patient to the hospital emergency room, the general practitioner (GP) often contacts the pulmonologist first. To reduce their workload, the pulmonologist is supported by a COVID-19 triage team. The triage team not only assesses the health situation, but also informs the GP about the consequences of hospitalization, encouraging them to consider, for example, the little or no visitors, loneliness, protective measures that make people unrecognizable and a possible transfer to another hospital at a great distance, even abroad, with the language problems that may arise. Informing the GP prevents false expectations and unnecessary admissions. This approach reduces the number of unnecessary admissions. The triage team consists of specially trained medical professionals. The triage team works with a standardized list of concerns that is carefully reviewed with the family physician. Furthermore, when a COVID-19 patient arrives at the ED, a transfer nurse discusses possible aftercare with the family. In a hopeless situation, it may also be palliative care, which makes hospital admission unnecessary. In the case of a hospital admission, discussing aftercare at an early stage helps reduce an unnecessarily long hospital stay.

#### 4.2.2. Non-Physical Consultations

**Figure 2:**
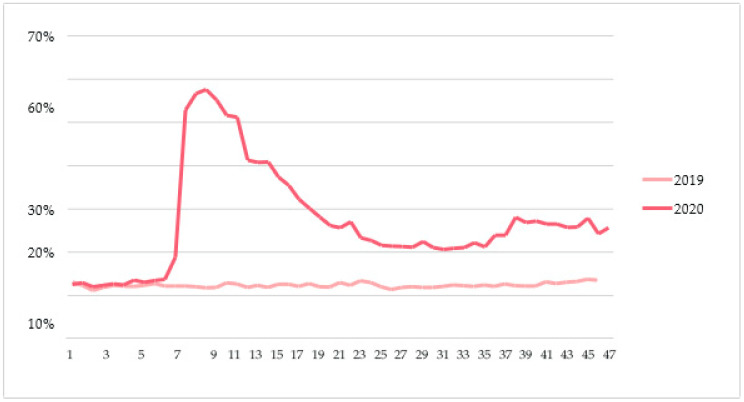
Relative number of non-physical consultations compared to the total number of consultations per week, for one of the hospitals [43].

All middle managers indicated that as a result of the COVID-19 pandemic, the number of telephone and online consultations increased at a rapid pace. Figure 2 shows, for one of the hospitals, the evolution of non-physical consultations over time.

The use of non-physical consults significantly relieved the pressure on outpatient clinics. Preoperative screenings (of ASA1 patients), intake interviews and control consultations were also handled in a non-physical manner in a number of hospitals. Despite the rapid increase in the use of non-physical consultations, it did present challenges both organizationally and in terms of a change in management in many cases. In this context, one of the managers told us that in some situations, face-to-face contact remains necessary and that when consulting online, some healthcare professionals are more likely to be distracted by “a doorbell ringing, a cat walking by or a child coming in”. One of the managers shared that some specialists were struggling with e-consults due to limited digital skills.

### 4.3. Increasing or Making More Adequate Use of the Available (Human) Capacity

#### 4.3.1. Increasing Responsiveness and Flexibility

##### Joint Decision Making Based on Up-to-Date Information

One of the managers explained that to increase the responsiveness and flexibility, there is a cross-disciplinary meeting every morning in which the following issues are discussed (based on up-to-date information):

1. The number of patients expected to be discharged before 2 pm (for each department).
2. Possible discharge bottlenecks and required actions.
3. The required admission capacity for the current and upcoming days.
4. Deployment of flexible staff capacity based on 1, 2 and 3 (see also the following subsection).

##### National Patient Distribution

To make more flexible use of the total available ICU capacity, ICU patients were dispersed across the country. To this end, a national coordination team was established, and national agreements were made. However, one of the middle managers indicated that the patient distribution was not always optimal, as not all hospitals provided full transparency on their available capacity. The reason for this was that various parties in the region tried to arrange their affairs outside of the central agreements, which led to ambiguity, irritation and even more work pressure.

#### 4.3.2. Making Use of Standardized Time Blocks

One of the managers indicated that, with the COVID-19 pandemic being the trigger, internal arrangements have been made with the various clinics and the Operating Room (OR) about the number of available places per day and per “time block”. The agreements are based on a quantitative analysis of historical data on the number of treatments per working day. This method reduced the uncertainty and improved the ability to anticipate (if necessary) in a more informed manner. And although this approach is not new, the COVID-19 pandemic prompted (at least for one of the hospitals) its introduction.

#### 4.3.3. Skill-Based Task Allocation

##### Deploying Non-Healthcare Employees

Furthermore, creativity and innovativeness were seen in deploying non-healthcare employees. By starting from the skills needed for a given task, for example, pool staff were used for supportive “care tasks”, and physiotherapists provided support in getting patients out of bed. According to one of the managers, ground stewardesses of a nearby airport were employed as hostesses in the hospital lobby. Also, nursing assistants and/or facility staff, students, secretary assistants and pensioners were deployed to provide support in the nursing wards with tasks like cleaning or being accommodating to visitors. Deploying non-healthcare employees reduced the pressure on the regular healthcare professionals.

However, it should be noted that not all managers were pleased with the creative employment of non-healthcare employees. The deployment of non-healthcare employees also required additional training time. For example, they first had to become familiar with some of the procedures. Some middle managers said that instructional videos were made for this purpose.

##### Deployment of Non-COVID-19-Related Specialists in COVID-19 Care

Specialists who saw their regular patient-flow “dry up” (such as oral surgeons and orthopedists) have been found to be willing to support lung specialists in their overloaded care agenda. For this purpose, in one of the hospitals, bootcamps were organized to provide these medical specialists with the required knowledge about COVID-19 care. After attending this bootcamp training course, they were able to triage new patients, answer questions from general practitioners or support the emergency department.

#### 4.3.4. Mental Support for Healthcare Professionals

Due to the COVID-19 crisis, many healthcare workers had to work under high pressure for a long time. From earlier studies, we know that a persistently high workload has a negative impact on the mental state of healthcare workers [44] and a negative impact on productivity [45,46]. In order to ensure that the inflow, throughput and outflow of COVID-19 patients proceeds as optimally as possible, it is therefore important to pay explicit attention to the mental state of hospital employees.

With the foregoing in mind, one of the involved hospitals proactively provided psychological and mental support, for example by deploying a psychologist during breaks and meetings in the COVID-19 departments. One of the managers brought up that they actively involved employees in establishing and modifying internal rules and guidelines in relation to the COVID-19 pandemic. The argument for this was that since the rules directly affect their work and changed almost weekly, it was important to give healthcare professional a say. One of the middle managers indicated that (for this purpose) they made a decision tree regarding COVID-19 procedures, which was available to all employees. The decision tree was modified based on the questions from and experiences of healthcare workers.

## 5. Discussion

The COVID-19 pandemic is considered the largest crisis since World War II and has put great pressure on hospitals. To handle the large and unforeseen influx of COVID-19 patients, a diverse range of actions and measures were taken, some of which focused on streamlining the inflow, throughput and outflow of patients. However, every hospital dealt with this exceptional situation in their own way. With the latter as a starting point, this paper provides an overview of actions and measures from various types of departments in a large number of hospitals in The Netherlands, based on interviews with nine expert and twelve middle managers.

Some actions and measures already existed before the COVID-19 pandemic. In hospitals in The Netherlands, admission to the ICU is already discussed with the patient. There is also a consultation with the GP as to whether a referral to the medical specialist is necessary. When a patient is discharged, the transfer nurse already talks with the family about potential aftercare options [47].

The study presented in this paper shows that a crisis situation also yields valuable (and generically applicable) improvements and innovations. Innovations in remote healthcare and eHealth applications and non-physical consultations that have already been initiated before the COVID-19 period have been further expanded [48]. Following the COVID-19 pandemic, a lot of hospitals are moving to a hybrid care approach that combines online non-physical care with personal care [49].

The interesting thing about some of the actions or measures identified is that they are also valuable (and can be applied) during non-crisis times. For example, joint decision making based on up-to-date information, making use of standardized time blocks and skill-based task allocation contribute to a more efficient use of capacity in hospitals. Given the current labor scarcity in The Netherlands and the burden on the nation’s meager financial resources for hospitals and healthcare, this is noteworthy. In the care of people with mental health issues, institutions want to focus on what employees can already do instead of on the degree previously obtained. These considerations offer flexible learning paths [50].

When it comes to lessons learned, the following can be concluded: First, alignment and collaboration are central to a majority of the identified actions and measures. In support of this, additional multidisciplinary coordination and consultation structures were set up, or already existing consultation meetings were used for this purpose. During these consultation meetings, decisions can be made quickly because (1) all key stakeholders are present and (2) up-to-date information is available. An adequate provision of information acts as a catalyst for good cooperation to respond quickly and adequately to a rapidly changing situation. People were happy with the short lines, the confidence that was created and the reduced bureaucracy. People have proved more willing to give up their own habits and interests. An important condition for the majority of the actions and measures described is openness to change and new ideas. In addition, the collaboration between middle managers as linking pins within and between care units and experts from support departments also turned out to be important. The added value of collaboration and coordination has become clear. Many forms of consultation both in the hospital and in the region that originated during the COVID-19 period have continued after this period in a less frequent form. The Dutch government believes that cooperation is essential to guaranteeing that healthcare is accessible, affordable and of good quality. The Integrated Care Agreement for 2022 contains agreements that this administration has made with a lot of stakeholders. Parties at the regional level develop regional strategies and pictures that outline their cooperation [51]. Collaboration when patient demand exceeds capacity without a crisis is not easy; it is like working against gravity [52].

Middle managers are important in assessing the feasibility of organizational measures for the primary care process proposed by experts and managers working outside the primary process. Some of the measures proposed by experts are difficult to implement for the middle managers in the care department. That is why middle managers sometimes opt for a second-best solution. During the exchange of personnel, it is important to consider employees’ own choice and to ensure good contact with them and attention to their well-being. Attention to their well-being appeared to be important to the choice of measures. For instance, middle managers chose to rotate employees in the COVID-19 department, which was not the best option, because a possible risk of infection exists. The attention to the well-being of employees is still important. Chronic stress reactions among healthcare professionals appeared to increase after the three COVID-19 waves [53].

Finally, we would like to note that we used a “hospital perspective”. Follow-up research, from the perspective of other healthcare parties (such as nursing homes and home care providers) could lead to insights into additional actions and measures.

## 6. Conclusions

In this contribution, we provide an overview of actions and measures for organizing and managing the inflow, throughput and outflow of COVID-19 patients within Dutch hospitals from various types of departments in a large number of hospitals in The Netherlands, based on interviews with nine expert and twelve hospital managers.

Based on the interview results, the following actions and measures have been identified: (1) improving ICU transfer and discharge, (2) COVID-19 cohort wards, (3) a COVID-19 triage tent with a CT scanner, (4) remote healthcare and eHealth applications, (5) adequate transition management, (6) non-physical consultations, (7) increasing responsiveness and flexibility, (8) making use of standardized time blocks, (9) skill-based task allocation and (10) mental support for healthcare professionals. These actions and measures can be categorized into three focus areas: streamlining of the underlying in- and external processes, reducing unnecessary or undesirable inflow of patients and increasing or making more adequate use of the available (human) capacity. It has been found that Little’s Theorem is a helpful paradigm for arranging the actions and measures from the interviews. The main lessons learned are the importance of integral tuning in the care process, giving up habits and self-interest, good information provision and the middle manager as a linking pin.

## Figures and Tables

**Table 1:** Overview of hospital types and positions of middle managers and experts.

	Type of Hospital/Organization	Middle Managers *	Patient Flow Logistics Experts **
1	Academic	Team leader, Healthcare manager	Advisor process improvement and implementation, Advisor business intelligence
2	Academic	Manager healthcare department	
3	Top clinical	Manager business (2)	Advisor integral capacity management (ICM)
4	Top clinical	Head of healthcare division, Head of outpatient department	Advisor project management
5	Top clinical	Manager	
6	General	Head of healthcare division (2)	
7	General	Manager business	
8	General	Team manager	
9	General		Head of division patient flow logistics
10	General		Capacity manager
11	General		Program manager operational excellence
12	Consultancy		Project leader capacity coordination healthcare
13	Consultancy		Consultant patient flow logistics

*: Interviews conducted in 2022–2023. **: Interviews conducted in 2020.

**Table 2:** Actions and measures.

Focus Areas	Actions and Measures
1. Streamlining of the underlying in- and external processes	Improving ICU transfer and discharge COVID-19 cohort wards COVID-19 triage tent with CT scanner Remote healthcare and eHealth applications
2. Reducing unnecessary or undesirable inflow of patients	Adequate transition management Non-physical consultations
3. Increasing or making more adequate use of the available (human) capacity	Increasing responsiveness and flexibility Making use of standardized time blocks Skill-based task allocation Mental support for healthcare professionals

## Data Availability

The data are not publicly available as the data are qualitative text data from interviews.
